# *Cryptococcus neoformans* infections: aspartyl protease potential to improve outcome in susceptible hosts

**DOI:** 10.1128/mbio.02733-24

**Published:** 2024-10-23

**Authors:** Frédérique Vernel-Pauillac, Christine Laurent-Winter, Laurence Fiette, Guilhem Janbon, Vishukumar Aimanianda, Françoise Dromer

**Affiliations:** 1 Institut Pasteur, Université Paris Cité, CNRS, Molecular Mycology Unit, UMR 2000, Paris, France; 2 Institut Pasteur, Université Paris Cité, CNRS UAR 2024, Mass Spectrometry for Biology Unit, Proteomics platform, Paris, France; 3 Institut Pasteur, Human Histopathology and Animal Models Unit, Paris, France; Texas Christian University, Fort Worth, Texas, USA

**Keywords:** *Cryptococcus neoformans*, aspartic protease Pep1p, vaccination, prophylaxis, passive therapy, monoclonal antibodies

## Abstract

Though a confined or a broad population is exposed respectively to endemic or pandemic infections, in the same environment, some individuals resist the development of infections. The attributed reason is the inheritance of a set of immune system genes that can efficiently deal with the pathogens. In this study, we show how outbred mice differentially respond to *Cryptococcus neoformans,* a fungal pathogen, and the mechanism through which the surviving mice mount a protective immune defense. We identified that those mice developing antibodies specifically against Pep1p, an aspartic protease secreted by *C. neoformans*, had significantly improved survival. Vaccination (either prophylactic or therapeutic) with a recombinant Pep1p significantly increased the survival of the mice by decreasing the fungal load and stimulating a protective immune response. Passive immunization of *C. neoformans-*infected mice with monoclonal antibodies developed against Pep1p also improves the survival of the mice by increasing phagocytosis of *C. neoformans* and decreasing the multiplication of this fungus. Together, these data demonstrate the prophylactic and therapeutic potentials of the *C. neoformans* antigenic protein Pep1p or Pep1p-specific antibodies against this fungal infection. Also, this study suggests that the immunological interaction and thereby the responses developed against a pathogen guide the hosts to behave differentially against microbial pathogenicity.

**IMPORTANCE:**

Vaccination and immunotherapies against fungal pathogens still remain a challenge. Here, we show using an *in vivo* model based on outbred mice that development of antibodies against Pep1p, an antigenic protein of the fungal pathogen *Cryptococcus neoformans*, confers resistance to this fungal infection. In support of this observation, prophylactic or therapeutic immunization of the mice with recombinant Pep1p could improve their survival when infected with a lethal dose of *C. neoformans*. Moreover, passive therapy with monoclonal anti-Pep1p antibodies also enhanced survival of the mice from *C. neoformans* infection. The associated antifungal mechanisms were mounting of a protective immune response and the development of fungal specific antibodies that decrease the fungal burden due to an increase in their phagocytosis and/or inhibit the fungal multiplication. Together, our study demonstrates (a) the mode of host–fungal interaction and the immune response developed thereby play a crucial role in developing resistance against *C. neoformans*; (b) Pep1p, an aspartic protease as well as an antigenic protein secreted by *C. neoformans*, can be exploited for vaccination (both prophylactic and therapeutic) or immunotherapy to improve the host defense during this fungal infection.

## INTRODUCTION

Cryptococcosis is associated with HIV infection and can occur in patients with other cellular immune defects, whether linked to a treatment such as prolonged steroid intake or diseases (malignant lymphoid diseases, organ transplantation, sarcoidosis, glucocorticoid treatment, chronic liver diseases, diabetes, etc.) or in patients with apparently no immunodeficiency (1, 2). Despite guidelines for the diagnosis and management of patients with cryptococcosis, this fungal infection still accounts for approximately 20% of deaths related to HIV infections (3, 4). *Cryptococcus neoformans* is now listed as a critically risky pathogen in the World Health Organization (WHO) fungal priority pathogens list with an advocacy to ending death from HIV-related cryptococcal meningitis by 2030 (https://www.who.int/publications/i/item/9789240060241).

The incidence of cryptococcosis and the outcome of the infection vary among susceptible hosts (HIV-infected or uninfected) and depend on geographical areas, underlying risk factors (5–7), central nervous system involvement, and timing of diagnosis and therapeutic management (2, 6–10). These studies lead to the query—why only a part of immunocompromised or apparently immunocompetent population develop cryptococcosis or cryptococcal meningoencephalitis, though they have the same underlying disease, risk factor, or even are from the same geographical areas? One of the hypotheses for some individuals remaining healthier than others is attributed to their optimal immune resilience, a capacity to preserve and rapidly restore immune function that offers resistance to infections (8). Development of novel adjunct therapies that could increase the efficacy of antifungal drugs, especially in the context of suboptimal immunity, may warrant evaluation. Supporting this hypothesis, earlier, we associated the kinetics of the antibody response during murine cryptococcosis with infection outcomes (11). Here, we further demonstrate that mice that recognize and respond to an antigenic protein of *C. neoformans,* Pep1p, develop resistance to this fungal infection. Confirming it, we show that immunological intervention (prophylactic or therapeutic immunization) with Pep1p as well as passive transfer of anti-Pep1p antibodies protect mice from a lethal challenge with *C. neoformans*.

## RESULTS

### Mice surviving longer after *C. neoformans* infection develop anti-aspartic protease antibodies

**Fig 1:**
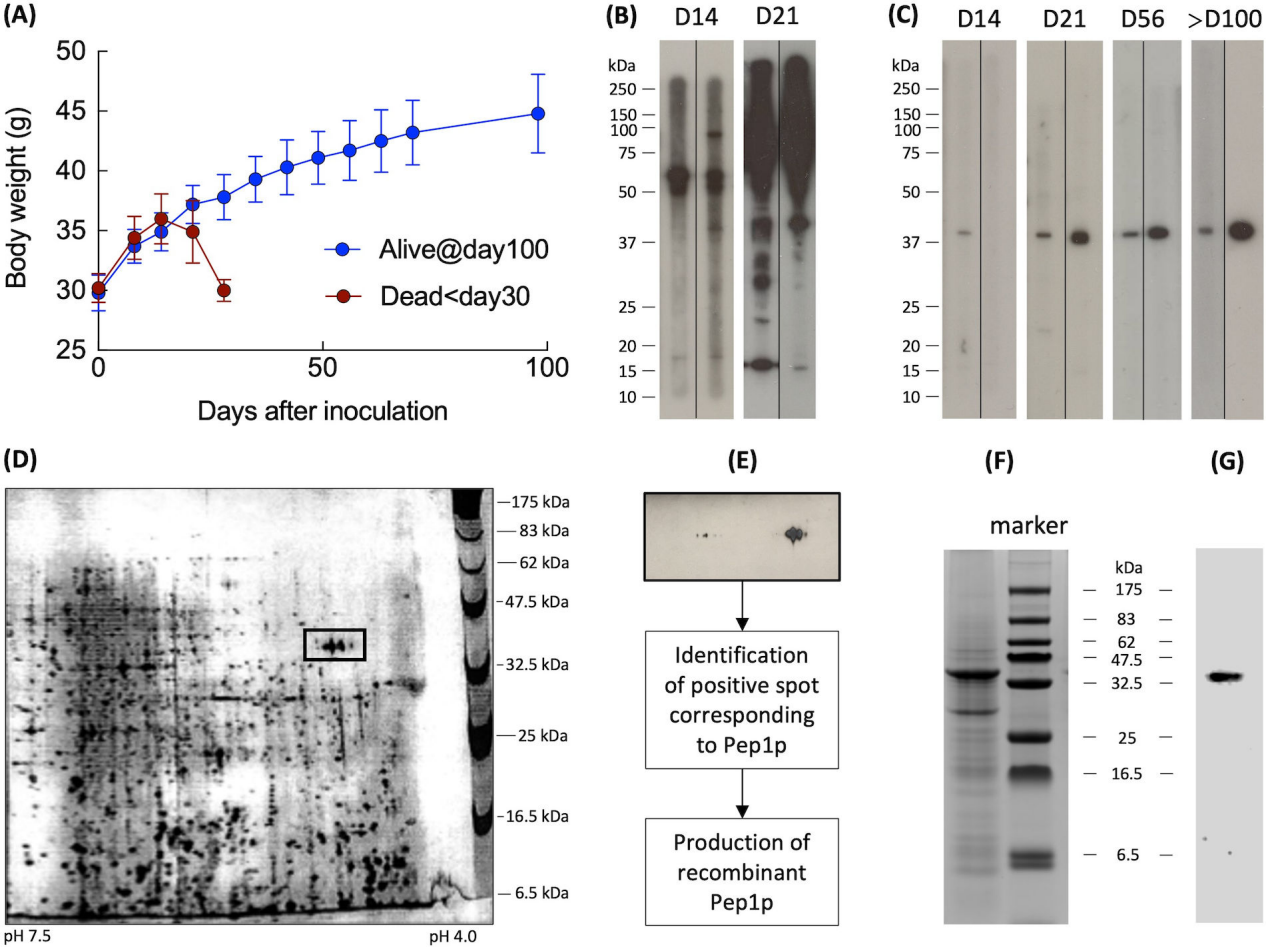
Development of anti-Pep1p antibodies is associated with prolonged survival in mice inoculated with *C. neoformans*. (**A**) Body weight evolution in mice (*n* = 28, two independent experiments) inoculated intravenously with *C. neoformans* NIH52D (10^3^ yeasts/mouse) that died within 30 days (non-survivor group) or survived >100 days (survivor group). Sera from two representative mice, (**B**) from the non-survivor group showed multiple bands on Western blot against the cytosolic fraction of *C. neoformans*, (**C**) while there was a single band with sera from two representative mice of the survivor group followed >100 days, (**D**) 2D-PAGE of the *C. neoformans* cytosolic fraction stained with Coomassie blue, (**E**) Western blot of the 32–47 kDa band cut from this 2D-PAGE with pooled survivor group sera, (**F**) SDS-PAGE profile of recombinant Pep1p (Coomassie silver staining), and (**G**) Western blot of rPep1p with pooled survivor group sera.

Outbred OF1 mice were infected with *C. neoformans* var. *neoformans* isolate NIH52D, with two doses – 10^3^ (*n* = 28, in two independent experiments) and 5 × 10^3^ yeasts per mouse (*n* = 14, in one independent experiment). We identified two behaviors following inoculation: early death within 30 days (non-survivor group; 50% survival for 10^3^ yeasts per mouse and 22% survival for 5 × 10^3^ yeasts per mouse) or prolonged survival over 100 days (survivor group, between 50% for 10^3^ yeasts per mouse and 22% for 5 × 10^3^ yeasts per mouse). The non-survivor group showed body weight loss from 14 dpi onward, whereas the survivor group showed a weight gain over time (Fig. 1A; showing data for inoculum size 10^3^ /mouse is presented). When probed against the cytosolic fraction of *C. neoformans* by Western blot, sera from mice of the non-survivor group showed multiple bands (Fig. 1B), and that from the survivor group mice resulted mainly in a single band at an apparent molecular mass of 40 kDa (Fig. 1C).

Following this, the cytosolic fraction of *C. neoformans* var. *neoformans* was subjected to 2D-PAGE, in duplicate. One gel was stained with Coomassie blue, and the other was subjected to immunoblotting using pooled serum of six mice from the survivor group (Fig. 1D and E, respectively). The spot corresponding to the positive immunoblot was excised from the Coomassie blue stained gel, processed, and subsequent analysis identified the protein as an aspartic protease, Pep1p (CAN_05650).

### Expression of recombinant Pep1p (rPep1p) is efficient in the *Escherichia coli* system

We then sorted to produce a recombinant Pep1p to explore its immunization potential, for which we tried different expression systems. Both *Pichia pastoris* and *Saccharomyces cerevisiae* expression systems failed to produce rPep1p. Therefore, we opted for the *E. coli* expression system with the vector pHAT10/11/12. rPep1p expressed on SDS-PAGE showed a major band at an apparent molecular mass of 40 kDa (Fig. 1F), but also showed several additional/degraded bands. However, Western blot using pooled serum from the survivor group showed a single positive band at 40 kDa (Fig. 1G), suggesting that additional and degraded bands resolved on SDS-PAGE could be due to auto-degradation and/or polymerization of Pep1p.

### Recombinant Pep1p is antigenic

**Fig 2:**
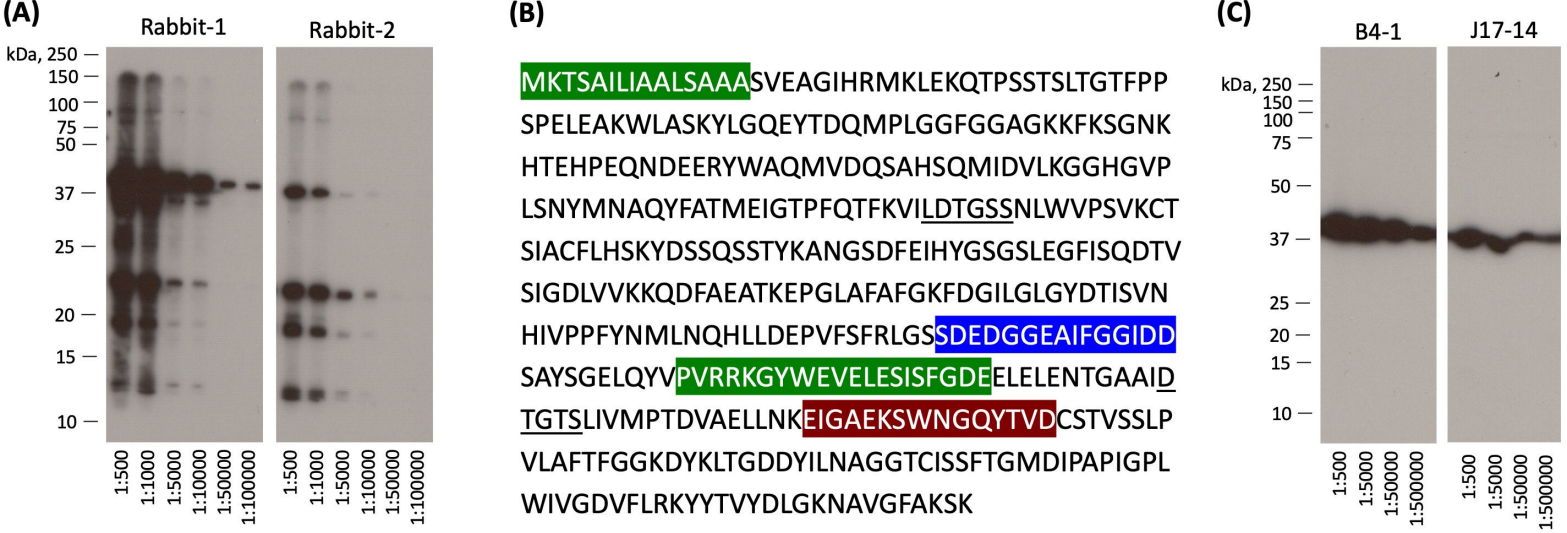
Characterization of polyclonal antibodies raised in rabbits and the monoclonal antibodies (mAbs) generated from mouse. (**A**) Sera from two rabbits immunized with rPep1p (500 µg rPep1p/rabbit; subcutaneously along with Freund’s adjuvant, thrice with 15-day intervals between immunization) were subjected to Western blot against rPep1p at different serum dilutions, and similar to the SDS-PAGE migration pattern of rPep1p (Fig. 1F), there were several positive bands indicating the polyclonal nature of the antibodies raised in rabbit against rPep1p. (**B**) Pep1p sequence and the epitopes recognized by the three clones of mouse monoclonal antibodies (mAbs) generated; in green, epitopes recognized by mAb J1-26; in blue, epitope recognized by mAbs J17-14 and B4-1; in brown, epitope recognized by B4-1. Underlined are the active site residues of Pep1p. These epitopes were conserved in the homolog aspartyl protease of *C. neoformans* var. *grubii*. (**C**) Western blot profiles showing that the mouse mAbs recognize rPep1p at different dilutions. Blots for B4-1 and J17-14 are presented; unlike Western blot profiles with rabbit polyclonal antibodies, only a band at an apparent molecular weight of 40 kDa was recognized by these mouse mAbs.

Recombinant Pep1p was used to immunize rabbit or mouse to generate polyclonal and monoclonal antibodies, respectively. Of note, none of the pre-immunized sera reacted with recombinant Pep1p when examined by Western blot. Rabbits immunized with recombinant Pep1p showed the presence of anti-Pep1p antibodies in their sera when examined by Western blotting against rPep1p. Like SDS-PAGE (Fig. 1F), the polyclonal rabbit antisera recognized multiple bands of rPep1p (Fig. 2A). On the other hand, three clones of mouse monoclonal antibodies (mAbs) were generated by the hybridoma technique: B4-1, J1-26, and J17-14. All these three mAbs were identified to be IgG1. By enzyme-linked immunosorbent assay (ELISA) and using non-overlapping as well as overlapping 15-mer peptide sequences, we could identify Pep1p epitopes recognized by these mAbs (Fig. 2B), which were all outside its enzymatic active site. Interestingly, mAbs J1-26 and B4-1 recognized two epitopes of Pep1p. We checked that these epitopes were conserved on the aspartyl protease of *C. neoformans* var. *grubii* (CNAG_00581) (https://www.ncbi.nlm.nih.gov/protein/OWZ59865.1). Moreover, these mAbs, unlike rabbit polyclonal sera, recognized only rPep1p of an apparent molecular weight of 40 kDa.

### Cryptococcal Pep1 expression occurs *in vivo* in the infected mice

**Fig 3:**
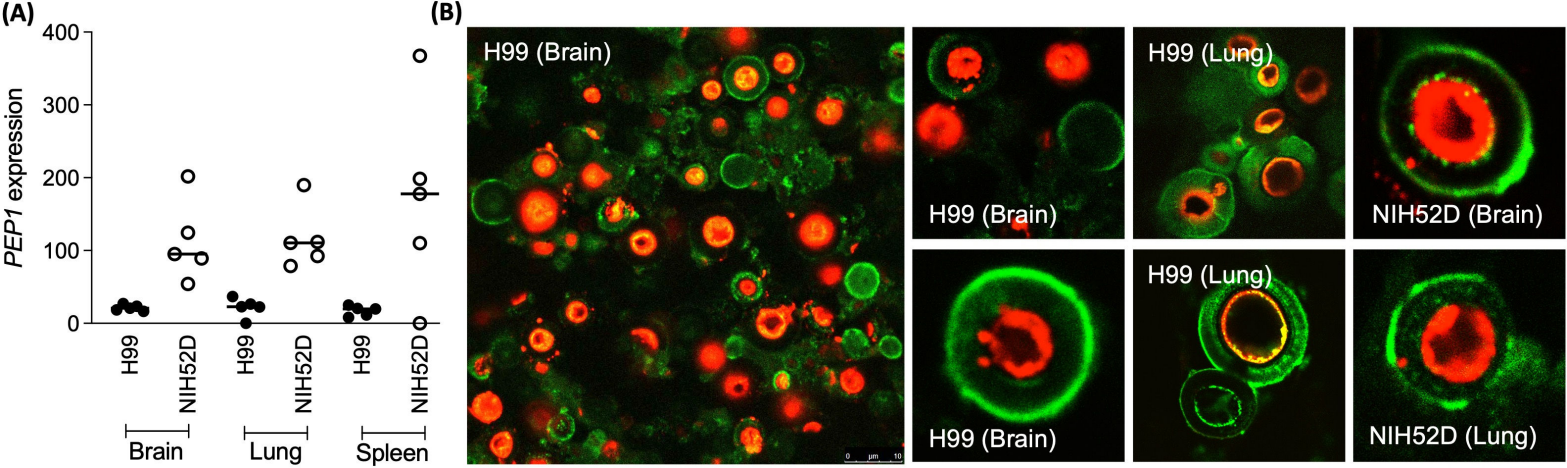
Expression of *PEP1* during murine infection. (**A**) *PEP1* expression in organs from mice intravenously infected with *C. neoformans* yeasts (H99 or NIH52D; 10^6^ per mouse) at 14 days post-infection (dpi); data are expressed as the copy numbers of *PEP1* relative to the fungal load in the indicated organ. Mean ± SD from a total of five mice for each fungal strain is presented. (**B**) Evidence for the secretion of Pep1 by *C. neoformans* (both H99 and NIH52D strains) during infection in the mouse brain and lung (paraffin section of the infected brain). The *Cryptococcus neoformans* capsule is immuno-stained with FITC-labeled E1 (anti-capsular polysaccharide antibody; green) and Pep1 (red) with polyclonal anti-rPep1 antibodies and TRITC-conjugated goat anti-rabbit IgG.

Quantitative PCR was performed to determine *C. neoformans PEP1* expression during experimental infection of mice in comparison with constitutive expression during the growth *in vitro* (stationary phase). Preliminary experiments indicated the threshold of detection to be 10^6^ colony-forming units (CFU)/g of the organ. *PEP1* was significantly more expressed in the brains of mice infected with the *C. neoformans* isolate NIH52D (10^6^ yeasts/mouse) compared to those infected with the H99 isolate (*C. neoformans* var. grubii, 10^6^ yeasts/mouse; *P* < 0.001), while fungal load was lower for the NIH52D isolate (log CFU/g brain = 6.4 ± .7 for NIH52D vs 7.4 ± 0.6 for H99) (Fig. 3A). The expression of *PEP1* showed the same trends in the other organs tested. Immunohistochemistry showed that Pep1p was secreted through the capsule during infection and was detected in the tissues surrounding the yeasts during both H99 and NIH52 infections (Fig. 3B).

### Immunization with rPep1p prior to *C. neoformans* inoculation improves survival of mice

**Fig 4:**
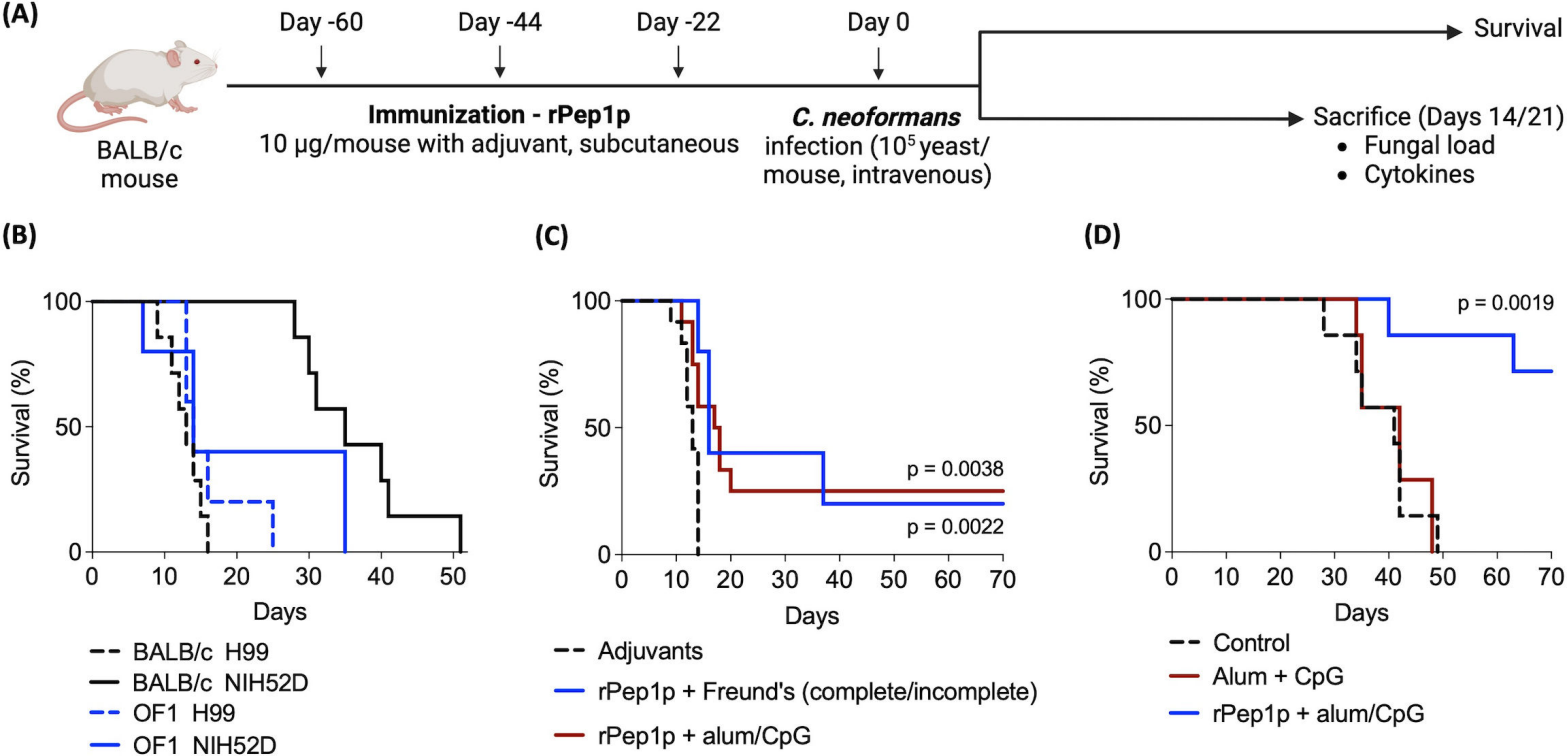
Prophylactic immunization with Pep1p increases survival of mice inoculated with *C. neoformans*. (**A**) Immunization with rPep1p, experimental design; rPep1p was administered subcutaneously, and *C. neoformans* yeasts (10^5^ /mouse; H99 or NIH52D) were inoculated intravenously. (**B**) Comparative outcome in BALB/c (total, *n* = 7 per group) and OF1 mice (*n* = 5 per group) infected with *C. neoformans* H99 or NIH52D strains (10^5^ yeasts/mouse) (one representative experiment). (**C**) Effect of adjuvants on the immunization efficacy of rPep1p; BALB/c mice infected with the H99 isolate. Control and immunization with rPep1p along with alum/CpG (12 mice/group, two independent experiments). Immunization with rPep1p and Freund’s complete and incomplete adjuvant (5 mice, one experiment). Survival data for immunization with rPep1*P* + alum or rPep1*P* + CpG are not presented; all the mice in these groups died before 20 days post-infection, like the control group. (**D**) Effect of rPep1p immunization on infection with NIH52D; seven BALB/c mice per group (one experiment). Statistical significance was determined by Kaplan–Meier survival analysis with the Mantel–Cox test.

Figure 4A represents the experimental setup. First, we analyzed the effect of the *C. neoformans* isolate (H99 vs NIH52D; 10^5^ yeasts/mouse) on the survival of BALB/c (inbred) and OF1 (outbred) mice. Regardless of the mouse strain, the H99 strain was more lethal than NIH52D (Fig. 4B). Next, we aimed at identifying the most efficient adjuvant for immunization with rPep1p. For this study, we exploited BALB/c mice and H99 as this strain was more virulent compared to NIH52D and thereby minimized the experimental duration. Immunization (subcutaneous route) was performed 60, 44, and 22 days before the inoculation with 10^5^ yeasts/mouse. The best regimen was the immunization with rPep1p along with alum and CpG, which significantly increased the survival of mice inoculated with H99 (Fig. 4C). Immunization with rPep1p and Freund’s complete and incomplete adjuvant formulation also prolonged the survival (Fig. 4C), while that with alum or CpG alone showed mortality similar to that of the group of mice injected only with the adjuvants (control group) (data not shown). Therefore, further immunization studies were performed with rPep1p along with alum and CpG in combination. With this formulation, we examined the immunization potential of rPep1p against the *C. neoformans* NIH52D isolate in BALB/c mice. As expected, given the difference in virulence between the two isolates (Fig. 4B), the improvement in survival following immunization was greater in mice infected with 10^5^ NIH52D (Fig. 4D). Altogether, these results suggested that the extent of virulence associated with different *C. neoformans* isolates plays an essential role in the efficacy of active immunization with Pep1p.

**Fig 5:**
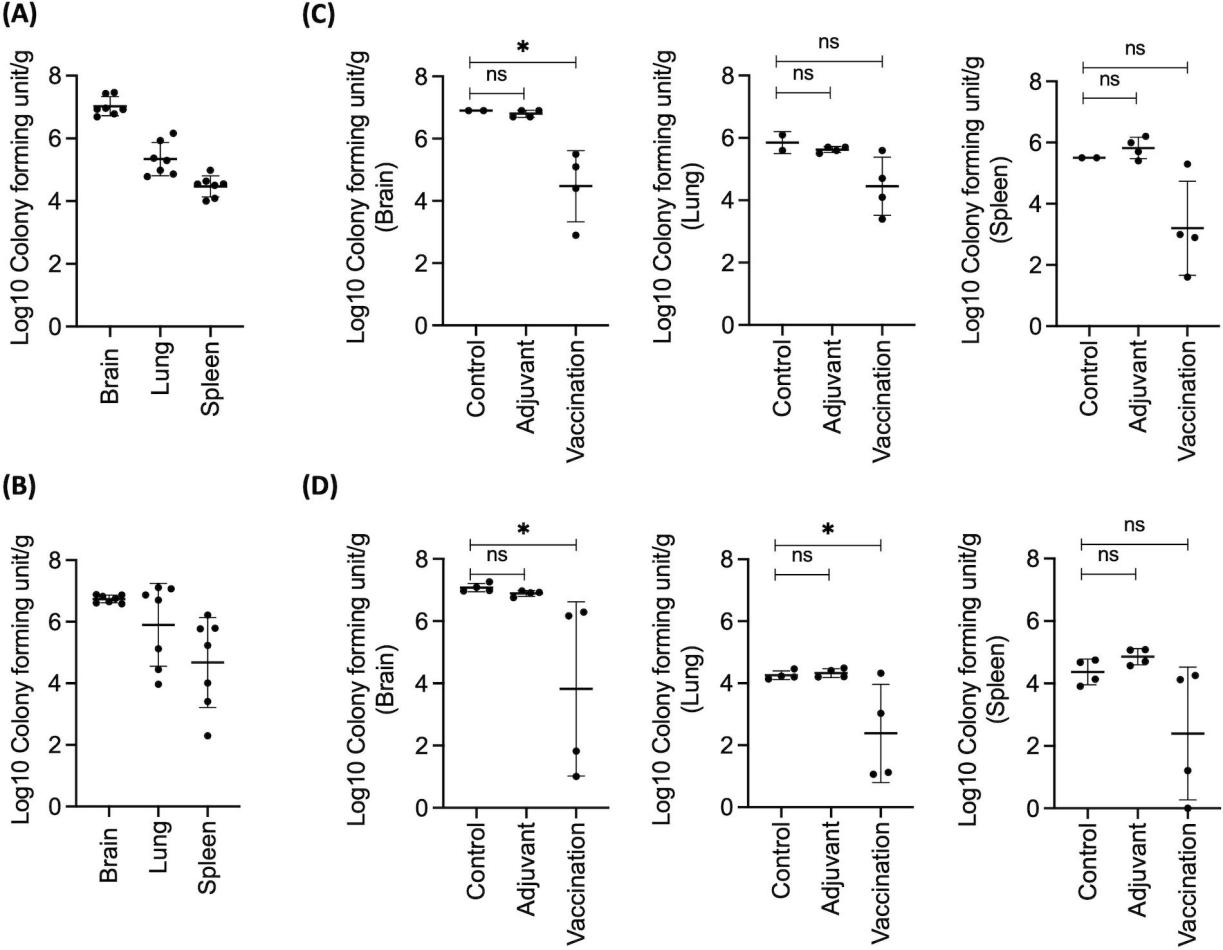
Immunization with Pep1p prior to inoculation with *C. neoformans* decreases the fungal burden in BALB/c mice. Recombinant Pep1p was administered subcutaneously along with alum/CpG; *C. neoformans* yeasts (10^5^ /mouse) were inoculated intravenously. (**A and B**) Colony-forming unit (CFU) per gram in the indicated mouse organs at 14 dpi; seven mice/group in two independent experiments; (**A**) H99 and (**B**) NIH52D. (**C and D**) CFU/g on day 14 in the indicated organs in mice immunized with rPep1p (+ alum/CpG) or injected with the adjuvant alone compared to control mice; four mice/group (one experiment), (**C**) H99 and (**D**) NIH52D (ns: nonsignificant, *P < 0.05).

We then quantified the fungal burden in the different organs (brain, lungs, and spleen) of mice infected with *C. neoformans* (Fig. 5A and B for H99 and NIH52D isolates, respectively) and in groups of mice immunized with rPep1p along with alum and CpG in combination prior to inoculation (Fig. 5C and D). Immunized (vaccinated) mice showed a significantly lower fungal burden in their brain compared to unimmunized mice or mice injected with the adjuvant, and this was observed for both H99- and NIH52D-infected mice. Compared to both controls, mice vaccinated with rPep1p also had a statistically significant reduction in the fungal burden in their lungs when infected with NIH52D, whereas the reduction did not reach significance for mice infected with H99 nor in the spleens after inoculation of both H99 and NIH52D. This study suggests that the magnitude of fungal burden reduction upon immunization may depend on the infecting strain and the organ.

**Fig 6:**
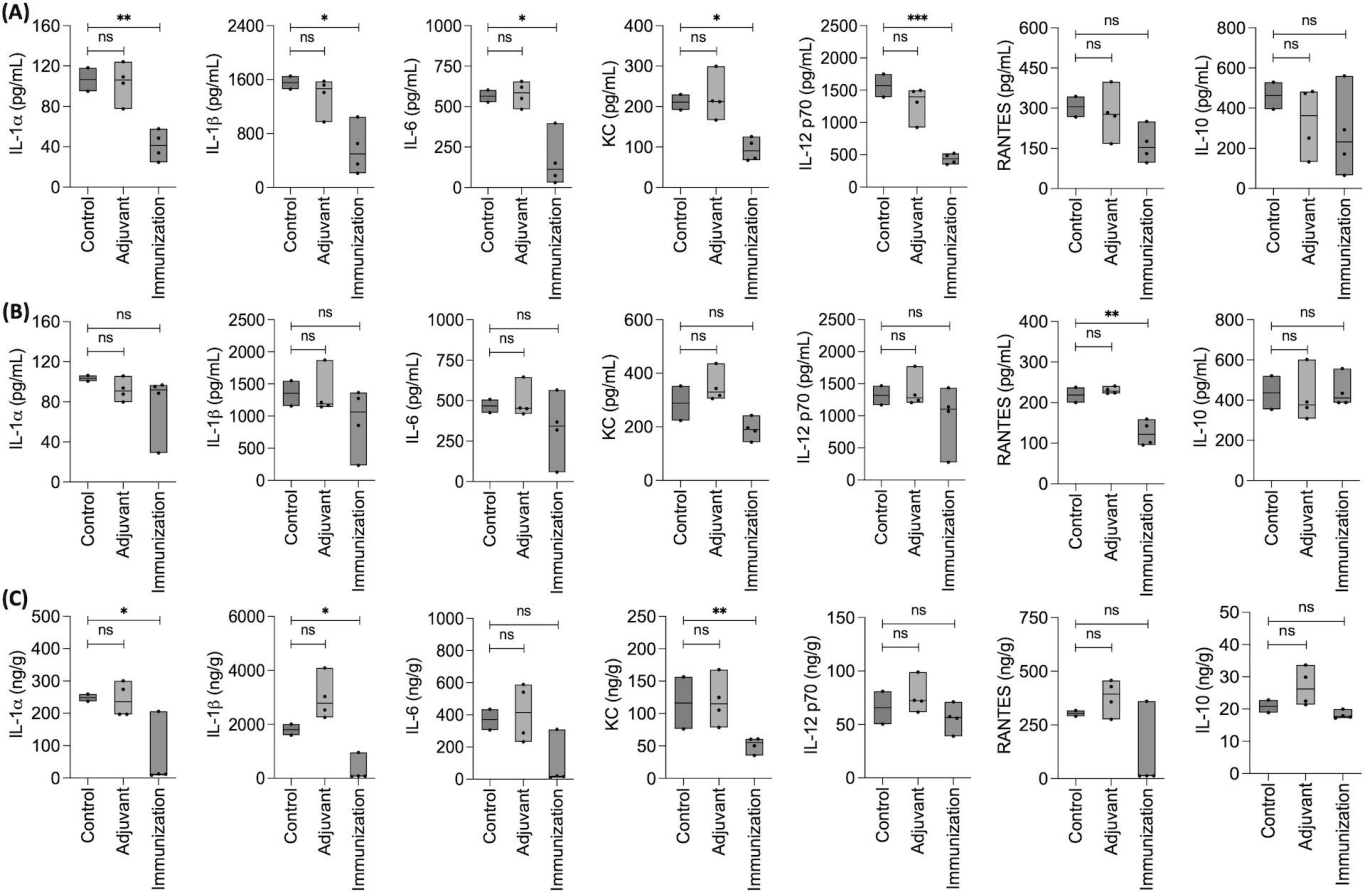
Prophylactic immunization with Pep1p modulates immune responses in mice. (**A and B**) Cytokine and chemokines in sera of rPep1p-vaccinated (subcutaneously) mice infected with *C. neoformans* (NIH52D; 10^5^ yeasts/mouse via intravenous route, two mice in control and four mice each in the adjuvant and immunization groups, in one experiment), compared to control mice and mice injected with adjuvants at 14 and 21 dpi, respectively. (**C**) Cytokines and chemokines in the brains in the same groups of mice; four mice in each group at 21 dpi. Statistical analysis was performed by one-way ANOVA with Dunnett’s multiple comparison test; ns: nonsignificant, *P < 0.05, **P < 0.005, and ***P < 0.0005.

Furthermore, we quantified cytokines and chemokines in the serum and brain homogenates of the three groups of mice (unimmunized, adjuvant injected, and rPep1p immunized) infected with *C. neoformans* (NIH52D isolate) at 14 and 21 days dpi. Immunization significantly decreased the levels of circulating cytokines and chemokines compared to unimmunized and adjuvant-injected groups of mice at 14 dpi (Fig. 6A) and only by a trend at 21 dpi (Fig. 6B). On the other hand, in the brain homogenates, immunized mice showed significantly lower levels of specific cytokines and chemokines at 21 dpi compared to unimmunized and adjuvant-injected groups (Fig. 6C). Together, these data suggest that immunization with recombinant Pep1p possibly reduces the hyperinflammation due to *C. neoformans* infection.

### Therapeutic immunization with rPep1p improves the outcome of *C. neoformans* infection

**Fig 7:**
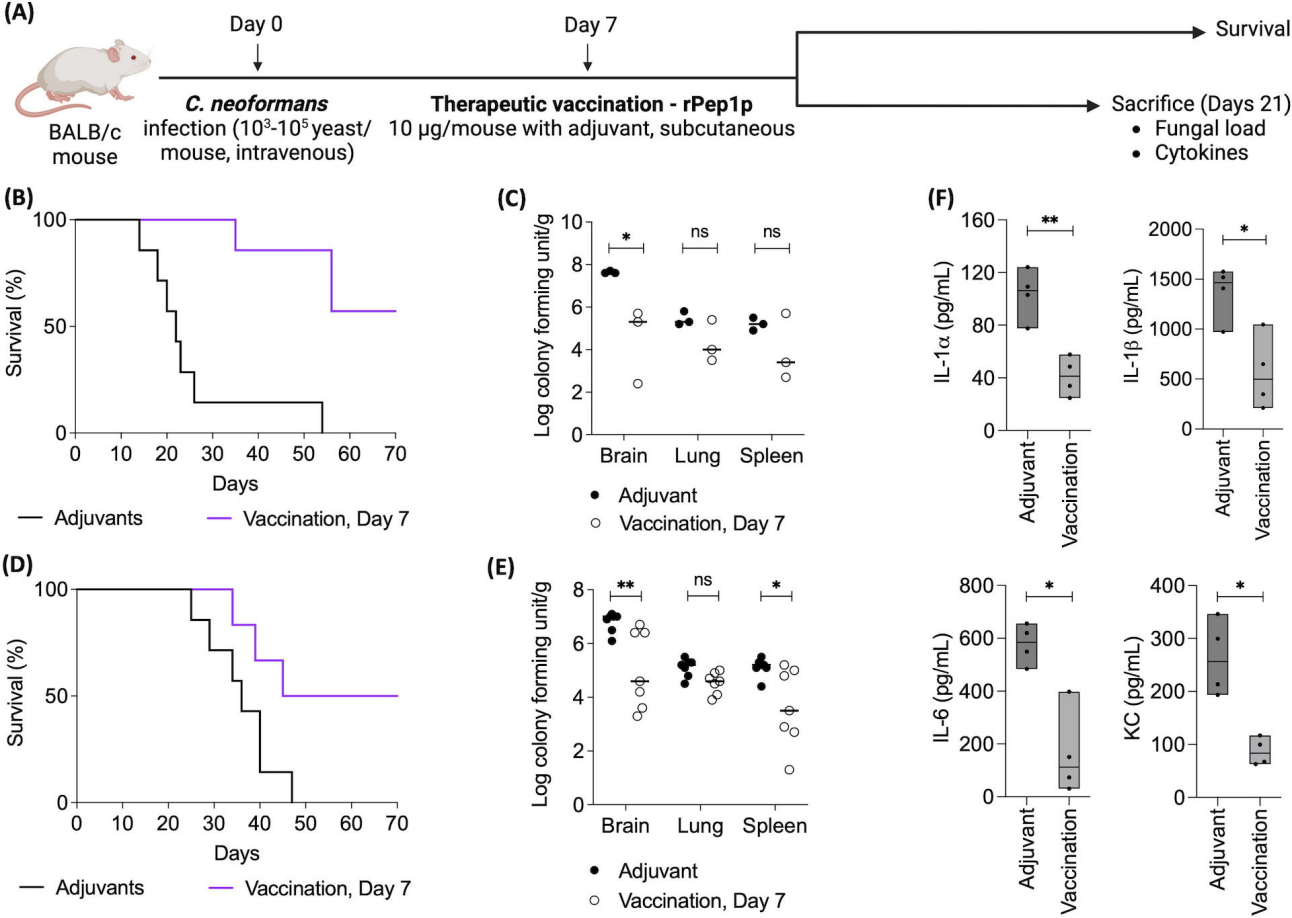
Therapeutic vaccination of mice with Pep1p improves the outcome of *C. neoformans* inoculation. (**A**) Experimental setup. (**B, D**) Survival of mice (*n* = seven mice/group, one experiment) intravenously infected with *C. neoformans* H99 (10^3^ /mouse) or NIH52D (10^5^ /mouse), respectively, and immunized (subcutaneously) with rPep1p at 7 dpi. (**C, E**) Log CFU/g in indicated organs of *C. neoformans* (H99 and NIH52D, respectively)-infected mice, injected with adjuvant or vaccinated on day 7 and sacrificed on day 21 (C – 3 mice/group, one experiment; E – 7 mice/group, two independent experiments). (**F**) Cytokine and chemokine levels in the serum of mice (4 mice/group, at 21 dpi, one experiment) injected with the adjuvant or vaccinated at 7 dpi. Statistical analysis was performed by one-way ANOVA with Dunnett’s multiple comparison test. ns: nonsignificant, *P < 0.05; **P < 0.005.

We then studied the potential therapeutic benefits of rPep1p injection on an already established *C. neoformans* infection in mice (Fig. 7; the experimental setup is presented in Fig. 7A). Injection with rPep1p of infected mice at 7 dpi significantly increased their survival compared to the group of mice injected only with the adjuvant, regardless of the *C. neoformans* strain used (Fig. 7B and D). Therapeutic immunization also significantly decreased the fungal burden in the brain and lungs of the infected mice euthanized 14 days after immunization compared to the group of mice injected only with the adjuvant (Fig. 7C and E). Moreover, immunization significantly reduced circulating (serum) cytokine and chemokine levels in the mice compared to adjuvant-injected mice (Fig. 7F; presented here is the data from mice infected with the *C. neoformans* H99 strain, immunized 7 dpi and subjected to cytokine and chemokine quantification 14 days after the immunization). Of note, of the seven mice infected with lethal dose of *C. neoformans*, four that survived had sterile brains. Together, therapeutic immunization with rPep1p increases the survival of mice infected with *C. neoformans* and reduced fungal burden and proinflammatory cytokine and chemokine levels compared to the group of mice injected with the adjuvant, suggesting that even therapeutic vaccination with recombinant Pep1p has beneficial protective effects against *C. neoformans* infection.

### Passive immunization with anti-Pep1p antibodies after *C. neoformans* inoculation improves the outcome of infection

**Fig 8:**
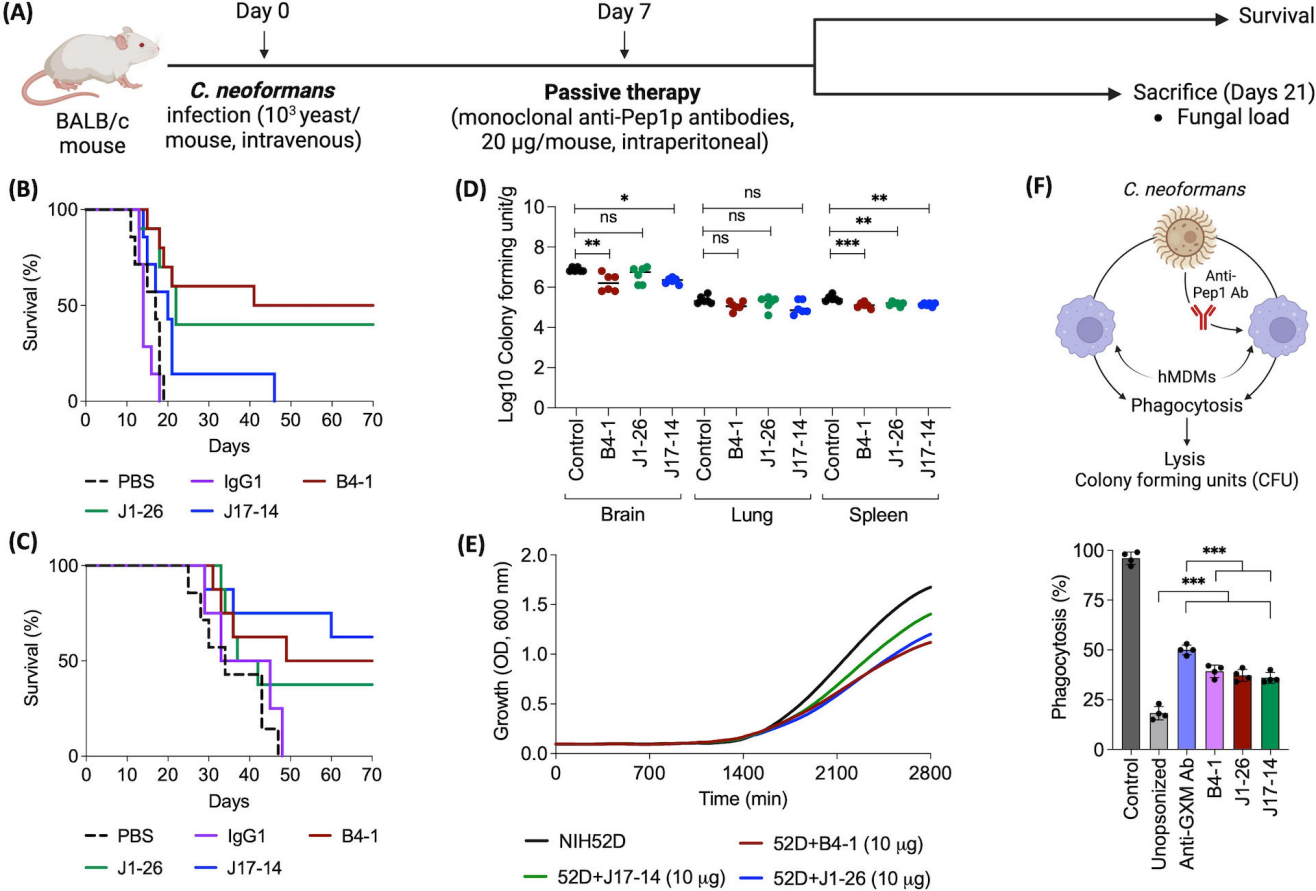
Administration of monoclonal anti-Pep1p antibodies following *C. neoformans* inoculation improves survival in the mice. (**A**) Experimental setup; monoclonal antibodies (mAbs) and *C. neoformans* were administered intraperitoneally and intravenously, respectively. (**B and C**) Survival of the mice infected with *C. neoformans* isolates H99 and NIH52D, respectively, and treated on 7 dpi with anti-Pep1p mAb (seven mice/group) (one experiment). (**D**) Log CFU/g in indicated organs of *C. neoformans-*infected mice (NIH52D) treated with anti-Pep1p mAb (five mice/group) (one experiment). (**E**) Growth of *C. neoformans* in the absence or presence of anti-Pep1p antibodies. (**F**) Phagocytosis of unopsonized or opsonized *C. neoformans* by hMDMs (*n* = 4); anti-GXM (**E1**) and anti-Pep1p (B4-1, J1-26, and J17-14) mAbs were used for opsonization. Statistical analysis was performed by one-way ANOVA with Tukey’s multiple comparison test; ns: nonsignificant, *P < 0.05, **P < 0.005, and ***P < 0.0005.

We analyzed the passive immunization potential of mouse monoclonal antibodies (mAbs) raised against rPep1p (clones B4-1, J1-26, and J17-14). The experimental setup is presented in Fig. 8A; an irrelevant IgG1 (chikungunya IgG1; CHIKV D3.62) was used as the negative control. Passive immunization with anti-Pep1p antibodies significantly increased the survival of the mice infected with either of the strains of *C. neoformans* (H99 or NIH52D), compared to control mice or mice injected with irrelevant IgG1. Although two doses (20 and 100 µg per mouse) of mAbs were tested for passive immunization, there was no significant difference in their levels of protection. Therefore, for further studies, the lowest dose (20 µg per mouse) of mAbs was used. The clones B4-1 and J1-26 resulted in comparable survival profiles regardless of the *C. neoformans* strain used for infection, whereas J17-14 resulted in strain-dependent improved survival of the mice (Fig. 8B and C). Mice injected with B4-1 or J17-14 showed a significant decrease in the fungal loads in the brain and spleen. J1-26 injection in mice resulted in reduced fungal load only in their spleens (Fig. 8D), while none of these anti-Pep1p antibodies affected fungal load in the mouse lungs.

We further dissected the possible mechanisms associated with these anti-Pep1p antibodies (B4-1, J1-26, and J17-14) in protecting mice from *C. neoformans* infection. *In vitro*, the multiplication of *C. neoformans* was significantly reduced in the presence of these anti-Pep1p antibodies (Fig. 8E). On the other hand, mAbs-opsonized *C. neoformans* yeasts were phagocytosed significantly better by the human monocyte-derived macrophages compared to unopsonized yeasts, even though less compared with anti-*C*. *neoformans* capsular (anti-GXM) antibody-opsonized yeasts (Fig. 8F). These data indicate that (i) anti-Pep1p antibodies inhibit the multiplication of *C. neoformans* yeasts and (ii) that opsonization by anti-Pep1p antibodies facilitates phagocytosis of *C. neoformans*.

## DISCUSSION

In this study, we observed that outbred mice showed a contrasted interaction with *C. neoformans* after intravenous inoculation. Mice that produced antibodies to a specific antigenic protein of *C. neoformans*, a secreted aspartic protease Pep1p, developed resistance to this fungal infection, whereas mice that show an antibody response to multiple antigens of *C. neoformans* developed an acute infection and died earlier after inoculation. On the other hand, BALB/c mice always died after inoculation, their survival being only a function of the inoculum size (the smaller the inoculum, the longer the survival) and the strain (longer survival with NIH52D than with H99 for the same inoculum size). Furthermore, we show that either active immunization with Pep1p, a recombinant aspartic protease, or passive immunization using anti-Pep1p antibodies significantly improves the outcome of *C. neoformans* infection in BALB/c mice. Active immunization (prophylactic as well as therapeutic) with Pep1p resulted in a decreased fungal burden in the murine organs (even complete eradication of the fungus in some mice) and a decreased inflammatory response. Passive immunization with monoclonal anti-Pep1p antibodies also conferred partial increase in the survival by reducing this fungal growth and facilitating the phagocytosis of *C. neoformans* via opsonization.

*Cryptococcus neoformans* is a biotrophic basidiomycetous yeast that is found ubiquitously in the environment. Most of us have been in contact with the fungus, as assessed by the presence of anti-cryptococcal antibodies in healthy individuals; evidence shows that when diagnosed, cryptococcosis followed reactivation of a latent infection (12, 13). This fungal pathogen can cause infection both in immunocompromised and immunocompetent hosts. However, not all vulnerable hosts will develop an infection (5–7, 9, 11, 14, 15), which is comparable to what we observed with outbred mice infected with *C. neoformans*. This could be attributed to two facts: (a) In an immunocompetent population, the genetic variations have been attributed to individual responsiveness to an invading microbe. An immune gene variant either confers resilience to fight against a pathogen or makes the host more susceptible to infection (8). (b) In immunocompromised patients, the extent of immune compromise could differ in each individual. Indeed, the attributed reason for cryptococcal meningoencephalitis in HIV patients is their low CD4 +T cell count (16). Individuals with acute-phase HIV infection and a proportion of HIV patients with clinical latency retain high levels of CD4 +T cell counts, which may be the cause for helping them combat *C. neoformans* infection (17).

Cryptococcosis still accounts for 19% of AIDS-related deaths in some countries despite awareness, available antigen testing, and antifungal treatment regimen based on amphotericin B, fluconazole, and 5-flucytosine (3, 18). However, even diagnosed and treated, cryptococcosis mortality remains high (19, 20). There have been several studies to develop vaccines against fungal infections, but none reached human application (10, 21, 22). The attributed reason is that fungal infection occurs mostly in immunocompromised hosts with low CD4 +T cell count (16), while studies rarely looked for vaccine development under immunodeficient conditions. We considered two different modes of immunization with recombinant Pep1p, prophylactic and therapeutic vaccination, and both protected immunocompetent mice from *C. neoformans* infection. It is understandable that prophylactic vaccination offers preparedness to those individuals who will be undergoing immunosuppressive interventions or therapies. Vaccination against a rare, even if life-threatening, non-transmissible infection may not be acceptable in terms of risk for the patient, but therapeutic vaccination in conjunction with appropriate antifungal therapy could be. Vaccination following an infection under immunosuppressive condition has been questioned for its efficacy and safety (23). However, although immunosuppressants modulate immune function through a control over CD4 +T cells, they only reduce the activation or efficacy but do not abolish immune system function (24). Therefore, recent studies suggest that the efficacy of antigens could be improved under immunosuppressive conditions either by customizing vaccine formulation with a robust adjuvant and efficient delivery system, increasing the dosage of vaccines, or through timed revaccination (25, 26).

Although vaccination started with live attenuated pathogens, the risk of reversion of attenuation led to explore potent antigenic components of these pathogenic microbes. While the nature of two well-explored antigenic molecules are proteins and polysaccharides (27), protein-based antigens have advantages over polysaccharide antigens; they are soluble, can be produced in recombinant forms, and they induce both cellular and humoral immunity (27). Moreover, unlike polysaccharide antigens that require their conjugation with carrier proteins to facilitate their transport across the target cell membrane, protein antigens can cross the cell membrane without carrier proteins. In addition, the protein-based antigens initiate T-cell-dependent B cell activation, a process that results in robust immune response; affinity maturation, leading to the production of immunoglobulins that will be pathogen-specific; and immunological memory (28). Aspartic proteases are identified as potential targets for antifungal therapies (29). A recombinant aspartyl protease has even been proven effective as a vaccine for murine coccidioidomycosis (30). Recent studies demonstrated that among the six proteins of *C. neoformans* (Cda1, Cda2, Cda3, Fpd1, MP88, and Sod1), expressed in recombinant forms, loaded into glucan particles and used to vaccinate mice, four could protect mice from lethal dose infection with *C. neoformans* (31), as did synthetic peptides derived from Cda2 (32). Development of the pulmonary inflammatory response and sterilizing immunity were the mechanisms upon vaccination that protected mice from infection. Interestingly, in this study (31), two strains of mice were used for vaccination, and they differed in their T helper cell epitopes upon vaccination. This suggests that the genetic background of the host plays a role in the immune response, which agrees with the observation of our study using outbred mice for recombinant Pep1p vaccination. However, in contrast with this study (31), we observed decreased cytokine/chemokine production, both in circulation and organs of the mice vaccinated with Pep1p. This could be due to the route of infection, orotracheal versus intravenous. Indeed, it is known that a dysregulated inflammatory response often worsens the fungal infection by limiting the protective antifungal immunity (33).

Adjuvants are indispensable during vaccination; they can function as immunopotentiators or delivery systems (34). Immunopotentiators lead to the activation and maturation of antigen presenting cells (APCs) and promotes the production of co-stimulatory molecules, thus enhancing the adaptive immunity, whereas delivery systems target antigens to APCs and increase the bioavailability of the antigen. In our study, we explored the adjuvant efficacy of the immunopotentiators (CpG, Freund’s adjuvant) as well as the delivery systems (Freund’s adjuvant, alum) for immunizing mice with recombinant Pep1p. We observed a significant increase in the survival of the mice against *C. neoformans* infection when they were immunized with Pep1p along with CpG in combination with alum, followed by Freund’s adjuvant. This suggests that the combination of CpG and alum increases the robustness of their properties in enhancing the antigenic potential of Pep1p. Indeed, alum-based adjuvants are approved for human application (35). On the other hand, though alum-based adjuvants induce a strong humoral response, they have been reported not to elicit a good cellular immune response and are associated with adverse effects at the site of administration (36, 37). In our study, we observed tissue necrosis and induration at the site of immunization when Pep1p was combined with Freund’s adjuvant.

When treatment options are either minimum, ineffective, or toxic, passive immunization (intravenous infusion of antibodies) is an alternative to prevent or treat a disease or infection. As fungal infections occur mostly in immunocompromised hosts with reduced cellular immune function, supplementation of specific antibodies with or without concomitant application of antifungal drugs is gaining attention as an efficient treatment strategy against fungal pathogens (38). The advantages associated with passive immunotherapy are (a) specific (monoclonal) antibodies (mAbs) can be raised against a wide range of fungal virulent factors (39); (b) mAbs provide immediate protection against systemic mycosis (40); (c) mAb-treatment avoids the emergence of antifungal resistance (40, 41); (d) synergism between mAbs and antifungal drugs will enhance the antifungal activity, thereby reducing the duration of antifungal treatment and their toxicity (40–42); and (e) mAbs will not alter the host microbiota (42). On the other hand, the disadvantages of passive immunotherapy are the cost to produce mAbs, their specificities demanding precise identification of the causative fungal pathogen before treatment, and their loss of efficacy during the progress of infection, which requires repeated infusion of mAbs at regular intervals (39–43).

We and others developed mAb against glucuronoxylomannan (GXM; a capsular polysaccharide and important virulence factor) of *C. neoformans* and demonstrated that injecting mice with anti-capsular mAb modifies the course of this fungal infection in a murine model (44, 45). The murine mAb, 18B7, was evaluated for its safety in HIV-infected patients treated for cryptococcal meningoencephalitis (46). 2G8 was another mAb developed against laminarin (a branched β−1,3-glucan oligosaccharide). The passive administration of 2G8 reduced the fungal load in the organs of mice infected a day before with *C. neoformans* (47); of note, β−1,3-glucan is a pan-fungal cell wall polysaccharide present even in *C. neoformans*. Two mAbs have been developed against melanin, an insoluble pigment that is produced by the DOPA (3,4-dihydroxyphenylalanine) pathway, present in the *C. neoformans* cell wall (48). Administration of these anti-melanin mAbs prior to inoculation with *C. neoformans* prolonged the survival of the mice. Put together, although mAbs have been successfully demonstrated to be effective against cryptococcosis, their production costs and the lack of funding arrested further development. Another issue is that these mAb treatments were performed prior to the inoculation, which makes their administration timing questionable in a real-life situation. Here, we showed that passive immunization was even efficient to reduce fungal burden and increase survival when the mAbs were administered in a host with an already established and otherwise lethal infection.

MAbs can exert antifungal activity by several mechanisms: by facilitating the phagocytosis of fungal pathogens via opsonizing them and/or by stimulating the release of cytokines that further modulate cellular immunity (42, 49). In immunosuppressed conditions with reduced cellular immune function, mAbs have been reported to exert direct antimicrobial activity as well as to modify the release of virulence factors from fungi (50–52). These studies suggest that mAbs can be effective against an infection both in immunocompetent and immunocompromised conditions. In agreement, in our study, we show that the anti-Pep1p antibodies can reduce the growth as well as increase the phagocytosis of *C. neoformans* by opsonizing their yeasts. On the other hand, secreted proteases of *C. neoformans* play a crucial role in virulence, and manipulation of their expression/activity has been considered to be a strategy to prevent infection. In this direction, neutralizing activity of secreted Pep1p could be an additional mechanism exerted by mAbs against *C. neoformans* infection but needs validation (53). The hybridoma technology available to date has eased the production of chimeric, humanized, and/or completely human mAbs (54, 55). Also, studies have advanced in converting non-fungicidal mAbs into fungicidal upon attaching a radiation emitter to them (40, 56). Moreover, the production of therapeutic mAbs will be lower than that of prophylactic mAbs, as in the latter condition, the etiological fungal agent causing infection has been already identified and one can produce the mAbs against well-known virulence factors of this pathogen (42).

Overall, our study uncovers two major aspects associated with *C. neoformans* infection: (a) immune susceptibility or resilience plays a crucial role in making the host vulnerable or resistant, respectively, to this fungal infection; (b) targeted interaction with *C. neoformans* and the development of a specific immune response against the fungal antigen Pep1p makes hosts resilient to this fungal infection. Unlike polysaccharide antigens (GXM/β−1,3-glucan oligosaccharide) or melanin, the protein antigen Pep1p should confer better/improved control over cryptococcosis. Also, in this study, we demonstrate that immunization with Pep1p (as either prophylactic vaccine or for therapeutic intervention once infection is already established) or injection of monoclonal anti-Pep1p antibodies improves the control of *C. neoformans* in infected mice, thereby making them potentially useful at various stages of this fungal infection process. Moreover, our hypothesis is that the pattern of specific antibodies combined with cytokine/chemokine detection in patients with cryptococcosis could help define the severity of infection. Selected patients could then benefit from specific therapeutic interventions with rPep1 or anti-Pep1p antibodies, in addition to conventional antifungal treatment.

## MATERIALS AND METHODS

### Fungal strains

*Cryptococcus neoformans* var. *grubii* (serotype A, *MAT*α, H99) and var. *neoformans* (serotype D, *MAT*α, NIH52D) were used in this study. Strains were stored at −80°C in 40% glycerol (stock). The yeasts were cultured from the stock on Sabouraud agar, sub-cultured in yeast extract–peptone–glucose liquid medium (YPD, Difco Laboratories, Detroit, MI, USA) for 22 hours at 30°C in a shaking incubator (150 rpm), collected by centrifugation, washed with phosphate-buffered saline (PBS), counted under a microscope using a hemacytometer, and suspended at desired counts in appropriate buffer/medium.

### Reagents

Unless otherwise specified, the reagents were purchased from Sigma-Aldrich. The alum (Alhydrogel 2%, an aluminum hydroxide wet gel suspension) was purchased from InvivoGen (San Diego, CA, USA). CpG-ODN1826 was purchased from VacciGrade (Cayla, Toulouse, France), a Class B oligonucleotide (5’-tccatgacgttcctgacgtt-3’) targeting Toll-like receptor-9 (TLR9) in mice. Conjugated antibodies were purchased from Bio-Rad Laboratories (Marnes-La-Coquette, France).

### Animals

Experiments were carried out in accordance with European Directive 2010/63/EU. The protocols were approved by the Hygiene and Safety Committee for Working Conditions (Protocol N^o^: 14.135) and the Institutional Ethics Committee in Animal Experimentation (No: 2013.0135). The mice in the experiments were monitored daily and were subject to the rules and controls of the Animal Welfare Structure established within the institute. Six-week-old outbred OF1 (Charles River, l’Arbresle, France) and BALB/cJRj (Janvier, Le Genest-St.-Isle, France) male mice were housed at a maximum of seven/cage in animal facilities; they received food and water *ad libitum*. To avoid selection bias related to their clinical condition, the mice euthanized on a given day were selected based on their tag previously assigned by a collaborator not involved in the project. Female New Zealand White rabbits, 12 weeks old, obtained from Charles River Laboratory were used to produce polyclonal antibodies.

### *In vivo* infection experiments

Mice were identified individually and infected with *C. neoformans* NIH52D or H99 strains (inoculum size specified in the *Results* section) intravenously. Viability of the yeasts was assessed by plating them on Sabouraud agar and counting the colony-forming unit (CFU). After inoculation, mice were either monitored daily (with weekly body weight recording and antibody response determination by collecting the blood sample from the lateral tail vein) or euthanized by CO_2_ inhalation at predetermined timepoints [with collection of blood for antibody detection by Western blot, cytokine/chemokine level determination by Luminex technology (Bio-Plex Pro Assay, Mouse Cytokine/Chemokine and Growth Factors, 23-Plex, Group I (BIO-RAD) and their organs for CFU determination, histopathology, and cytokine/chemokine level quantification.

### Identification of proteins triggering antibody responses in mice surviving a lethal challenge with *C. neoformans*

The cytosolic fraction of *C. neoformans* strain NIH52D was prepared as described before (11). Aliquots of the cytosolic fraction were subjected to 12% sodium dodecyl sulfate-polyacrylamide gel electrophoresis (SDS-PAGE), followed by Western blot using pooled sera from mice from the non-survivor group or the survivor group. The *Cryptococcus neoformans* cytosolic fraction was also subjected to two-dimensional (2D) PAGE, with a first step of isoelectric focusing and a second dimension run in a 12% gel. Two identical gels were run at a time: one was used for immunoblotting and the other stained with Coomassie blue. Immunoblotting was performed using pooled sera from the survivors’ group. Proteins from the gel were transferred to Immobilon-P membranes (Millipore, France) for 30 minutes at 15V using a semi-dry electrophoretic transfer cell (Bio-Rad, Marnes-La-Coquette France). The membranes were subsequently blocked using TBS (50 mM Tris pH 8.0, 0.15M NaCl, 0.1% Tween-20) containing 5% non-fat milk (TBSTR) overnight at 4°C. Immunoblotting was performed with a multi-screen apparatus, using pooled mice serum (1:100 dilution in TBSTR) for 1 hour at room temperature, followed by the addition of horseradish-conjugated anti-mouse IgG (Bio-Rad, Marnes-La-Coquette, France) and a chemiluminescent substrate (Pierce ECL detection reagent, Thermo Scientific, Rockford, USA) for revealing. Spots of interest were excised from the 2D-stained gels, and in-gel tryptic digestion was performed as described previously with minor modifications using a DigestPro MSi robot (Intavis Bioanalytical Instruments AG, Germany). Briefly, after several washing steps of gel slices, proteins were reduced and alkylated. Enzymatic digestion was performed overnight with trypsin (Sequencing Grade, Promega, Madison, WI, USA). Digestion was stopped by adding formic acid. Peptides were further extracted, dried down, and resuspended in 12 µL solvent A (H_2_O: acetonitrile: FA; 98:2:0.1) prior to mass spectrometry (MS) analysis.

Peptides were analyzed on an LTQ-Orbitrap Velos instrument (Thermo Fisher Scientific, Bremen) equipped with a nano-HPLC Ultimate 3000 system (Dionex, Amsterdam, The Netherlands). Six microliters of each sample was loaded on a C_18_ trap column (300 µM inner diameter ×5 mm; Dionex), and peptides were further separated on an in-house packed 15-cm nano-HPLC column (75 µm inner diameter) with C_18_ resin (3 µM particles, 100 Å pore size, Reprosil-Pur Basic C_18_-HD resin, Dr. Maisch GmbH, Ammerbuch-Entringen, Germany). Sample loading was performed with a flow rate of 30 µL/minutes during 5 minutes, and then a flow rate of 300 nL/minute was used for peptide separation on the analytical column. A 40-minute gradient was used with the following conditions: 5 minutes 4% solvent B (H_2_O: acetonitrile: FA; 20:80:0.08), 4%–40% solvent B within 15 minutes, 40%–95% solvent B within 0.1 minutes, 95% solvent B for 5 minutes, 15 minutes 4% solvent B. The instrument method for the LTQ-Orbitrap Velos was set up in the data-dependent acquisition mode. After a survey scan in the Orbitrap (resolution 60,000), the 20 most intense precursor ions were selected for CID (collision-induced dissociation) fragmentation in the ion trap. The normalized collision energy was set up to 35 eV during 10 ms. The minimum signal threshold for triggering an MS/MS event was set to 5,000 counts. For internal mass calibration, the 455,120025 ion was used as lock mass. Charge state screening was enabled, and precursors with unknown charge state or a charge state of 1 were excluded. Dynamic exclusion was enabled for 90 seconds. Raw files were processed with Mascot v.2.4.1 as the search engine on Proteome Discoverer version 1.4.0.288 (Thermo Fisher Scientific) against a JEC21 genome database (Broad Institute) concatenated with known contaminants and reversed sequences of all entries. Trypsin was chosen as a specific enzyme with a maximum number of two missed cleavages. Possible modifications included carbamidomethylation (Cys, fixed) and oxidation (Met, variable). Mass tolerance for MS was set to 10 ppm, and 0.5 Da was used for MS/MS. Probability assignment and validation were performed using Scaffold software (version Scaffold_ 3.5.1, Proteome Software Inc., Portland, OR). A false discovery rate of 1% was used for both peptide and protein identification.

### Production of the recombinant protein Pep1p

*C. neoformans* var. *neoformans* were harvested after growing them to 5 × 10^7^ yeasts/mL in YPD. RNA was extracted with TRIzol reagent (Invitrogen, Carlsbad, CA) following the manufacturer’s instructions. Full-length cDNAs were obtained using the SMART 5’RACE cDNA amplification kit (Clontech) following the manufacturer’s instructions. The primers specific for the aspartyl protease (Pep1p) coding gene of *C. neoformans* var. *neoformans* were used to PCR amplify Pep1 cDNA. The PCR product was cloned using the TOPO vector following the manufacturer’s instructions. The construct (10 ng) was then transformed into BL21 Star(DE3) One Shot cells. Recombinant protein was expressed in their mature extracellular form (without signal peptide). The presence of poly-histidine (6xHis) tag in the pET TOPO allowed purification of the recombinant fusion protein (rPep1p) with a metal-chelating resin after solubilization of the inclusion bodies using 8 M urea. The purity of rPep1p was determined by running this recombinant protein on 12% SDS-PAGE, excising the protein bands, and then subjecting them to proteomic (mass and sequence) analysis.

### Anti-rPep1p antibody detection and production

Anti-rPep1p antibodies (Abs) were analyzed by ELISA. Briefly, rPep1p was coated overnight at 4°C (1 µg/mL in carbonate buffer) on a 96-well plate (MaxiSorp, Nunc, Roskilde, Denmark). Following this, wells were blocked with 1% gelatin in PBS for 1 hour at 37°C and washed with 0.1% Tween-20 in PBS (PBST). Samples diluted in PBST containing 0.25% gelatin (PBSTG) (100 µL/well) were incubated for 2 hours at 37°C (or overnight at 4°C). Specific antibodies were detected after incubation with the HRP conjugate (Bio-Rad) mouse IgG, followed by the addition of *o*-phenylenediamine as the substrate (SIGMAFAST). The optical densities (OD) were measured at 492 nm in a microplate reader (LabSystems Multiskan RC; ThermoFisher Scientific).

Polyclonal anti-Pep1p serum was produced by subcutaneously immunizing female New Zealand rabbits with rPep1p (500 µg) emulsified in Freund’s complete adjuvant (once, vol/vol) and Freund’s incomplete adjuvant (twice at 2-week intervals). Monoclonal antibodies (mAbs) were produced after fusion of spleen cells from BALB/c mouse immunized with rPep1p (10 µg/mouse, subcutaneously with alum, immunized thrice at 15-day intervals) and murine myeloma cells (P3 × 63-Ag8653) at a 1:4 ratio in 45% polyethylene glycol 1000. Three clones (B4-1, J1-26, and J17-14) were selected, and the mAbs were purified from ascites by ammonium sulfate precipitation. In all cases, production of anti-Pep1p Abs was tested by ELISA and Western blot.

Non-overlapping and overlapping peptides (15-mer) were synthetized (>90% purity, ProteoGenix, Schiltigheim, France) to analyze the epitopes recognized by the three mAbs produced. Peptides were suspended in PBS (5 mg/mL), and aliquots were stored at −20°C until further use. Peptides at various concentrations (1,250, 750, 250, 50, or 25 µM) were incubated for 1 hour at 37°C with the mAbs (5 ng/mL in PBSTG) and then processed for ELISA as described for the detection of anti-rPep1 Ab. Controls consisted of bovine serum albumin (25 µg/mL) in buffer (negative controls) and rPep1p (50 µg/mL, positive control). Results were expressed as percent inhibition upon incubation with peptides.

### Quantitative real-time PCR

Quantitative real-time PCR (qPCR) was performed to determine the expression of various genes of interest during experimental infection or *in vitro* growth. For the *in vivo* evaluation, whole organs from infected and non-infected mice were ground in 1 mL cold UltraPure DNase/RNase-free distilled water (UPW, Invitrogen, Carlsbad, CA), and 500 µL of the homogenate was washed in UPW +0,05% SDS (Sigma-Aldrich) at 4°C before sequential treatments with TURBO DNase, RNase Cocktail, and SUPERase-In (Ambion, Austin, Tx) to eliminate tissue debris. For the *in vitro* assessment, suspensions of yeasts recovered from cultures were standardized to 10^7^ per condition and pelleted. All pellets were immediately frozen in liquid nitrogen. After addition of β-mercaptoethanol (1:100 in 600 µL RTL lysis buffer, Qiagen), samples were homogenized (30 s at 7,000 rpm) twice with the Magna Lyser apparatus (Roche Applied Science). RNA extraction (RNeasy minikit, Qiagen) was followed by cDNA synthesis from 100 ng of DNase-treated RNA using the Transcriptor First-Strand cDNA Synthesis Kit and random hexamers (Roche Applied Science). All RNAs were processed simultaneously and in duplicate.

Primers were designed with the LightCycler Primer Probe Design Software 2.0 (Roche Applied Science) according to intron spanning and GC% (listed in Table 1). Standard curves for each target gene consisted in serial DNA dilutions (10^7^ to 1 copies). The LightCycler 480 II (Roche Applied Science) and the LightCycler 480 SYBR Green I Master kit were used. Reactions were carried out in duplicates (20 µL final volume, 0.5 µM primer, 2 µL DNA) with the following parameters (95°C for 10 minutes, followed by 45 cycles −95°C for 5 seconds, 60°C for 5 seconds with an extension time at 72°C depending on the size of the amplicon (1 s/25 bp). Absolute quantification of each target was done using the comparative cycle threshold (CT) method. The relative expression was calculated as the ratio of the gene target to the reference gene (glyceraldehyde 3-phosphate dehydrogenase, GAPDH) mRNA copy number (relative expression *in vivo*). Data are presented as the ratio of the relative expression *in vivo* to the relative expression *in vitro* measured (expressed as fold-increase *in vivo*/*in vitro* = (relative expression *in vivo*/*in vitro*) x 100). A further normalization was done to take into account the corresponding fungal load in target organs according to the following formula: fold-increase *in vivo*/*in vitro* divided by log CFU/g) x 100. Controls included non-infected mice for which PCR was negative.

**TABLE 1:** List of primers used for the study of the gene expression in *C. neoformans*

Primers (ID)	GenBank access	Sequence 5’−3’	Amplicon size (pb)	Tm (°C)
FVP/ACT-F1 FVP/ACT-R1	U_10867	CCA GAT CAT GTT CGA GAG TTT CA TCG ATA CGG AGG ATA GCG TG	177	85.5
FVP/ACT-F2 FVP/ACT-R2	XM_566845	AGA TCA TGT TCG AGA CTT TCA AT TCG ATA CGG AGG ATA GCG T	177	78
FVP/ACT-F3 FVP/ACT-R3	XM_566845	CCA CAC TGT CCC CAT TTA CGA CAG CAA GAT CGA TAC GGA GGA T	65	80.8
FVP/Pep1-F1 FVP/Pep1-F2 FVP/Pep1-R1	CNAG_00581	GGT TCG TCT AAT CTT TGG GT ATC CTG AGA GAT AAA GCC CT ATC CTG AGA GAT AAA GCC CT	162 159	84 83.5
GAPDH-F2231 GAPDH-R2427	XM_012195397	TGA GAA GGA CCC TGC CAA CA ACT CCG GCT TGT AGG CAT CAA	197	86.4
GAPDH/52D-F GAPDH/52D-R	XM_769908	CGA GAA AGA CCC TGC CAA CA ACT CCG GTT TGT AGG CAT CGA	197	86.6

### Efficacy of recombinant Pep1p immunization prior to or after challenge with *C. neoformans*

Preliminary experiments were performed to determine the best adjuvant for rPep1p immunization in four groups of five BALB/c mice receiving subcutaneously a mixture of rPep1p (10 µg) and one of the following: (i) Freund’s complete/incomplete adjuvant, (ii) Alum (2%), (iii) CpG (10 µg/mouse), and (iv) a mixture of 5 µg CpG and alum with boosts 14 and 28 days later. Controls were injected with saline mixed with the adjuvant. Following this, mice were infected with *C. neoformans* on Day 60 (10^5^ yeasts/mouse) and their survival monitored. Experiments were also performed to test the efficacy of rPep1p as a therapeutic vaccine by injecting *C. neoformans-*infected mice at 7 dpi with 10 µg rPep1p along with 5 µg CpG and alum (test group) or saline containing 5 µg CpG and alum (control). Details (strain, inoculum size, day of sacrifice, and number of animals) are specified in the relevant section of the *Results*. All injections were performed at the same time when endpoints included assessment of survival and fungal burden at selected time points.

### Anti-Pep1p mAbs in passive immunotherapy after challenging with *C. neoformans*

The effect of passive serotherapy with anti-rPep1p mAbs was first assessed with mAb B4.1 at 20 or 100 µg in PBS, injected intraperitoneally (ip) at 7 dpi with *C. neoformans* (10^5^ yeasts/mouse). Subsequent experiments compared the efficacy of B4-1, J1-26, and J17-14 (20 µg/mouse, ip) injected 7 dpi after *C. neoformans* infection (intravenous). The control group consisted of infected mice injected only with PBS. Both control and test groups of mice were monitored for their survival and fungal load.

### Efficacy of anti-Pep1p mAbs, *in vitro* assays

#### (a) Fungal growth

*C. neoformans* yeasts (10^4^/well) suspended in YPD broth with or without monoclonal antibodies (10 µg/well) were cultured, and their growth at time intervals was monitored by measuring the optical density at 600 nm (Bioscreen C MBR, ThermoFisher Scientific). The assay was performed in two independent experiments (biological replicates).

#### (b) Phagocytosis

Blood samples were collected from healthy donors from Etablissement Français du Sang Trinité (Paris, France) with written and informed consent, as per institutional ethics committee guidelines, Institut Pasteur (convention 12/EFS/023). Peripheral blood mononuclear cells (PBMCs) from blood samples (number of donors = 4) were isolated by a density gradient separation of Ficoll 400 (Eurobio, France). Isolated PBMCs were suspended in RPMI medium and seeded into 96-well culture plates (100 µL/well, containing 4 × 10^6^ cells/mL) and differentiated into macrophages (57). These monocyte-derived macrophages (hMDMs) were added with opsonized *C. neoformans* (1 × 10^6^ yeasts/well, opsonized with 5 µg of each mAb) and incubated at 37°C in a CO_2_ chamber for 1 hour. Following this, the culture supernatants were discarded, wells were washed with RPMI, and incubated further for 1 hour. The culture supernatants were discarded, hMDMs were lysed with cold water (500 µL/well), lysis was ensured by microscopic examination, and the lysates were collected. Each well was then washed twice with water (250 µL per well, each time), and the washings were added to the lysates. After appropriate dilutions of the collected lysates, aliquots were spread on Sabouraud agar plates, incubated at 37°C for 48 hours, and the *C. neoformans* colonies were counted. Unopsonized *C. neoformans* incubated with hMDMs were also processed like opsonized *C. neoformans*, while yeasts (1 × 10^6^) alone diluted like *C. neoformans* interacted with hMDMs and spread on Sabouraud agar plates served as the control. The positive control was the yeasts opsonized with anti-capsular monoclonal antibody E1 (44). The ratio between the colony-forming units for each condition compared to initial yeast count was presented as the percent of *C. neoformans* yeasts phagocytosed by hMDMs.

### Statistical analysis

Performed using GraphPad Prism software Version 10, and a *P*-value of <0.05 was considered statistically significant.

## References

[1] Maziarz EK, Perfect JR. 2016. Cryptococcosis. Infect Dis Clin North Am 30:179–206. doi:10.1016/j.idc.2015.10.006 26897067 PMC5808417

[2] Meya DB, Williamson PR. 2024. Cryptococcal disease in diverse hosts. N Engl J Med 390:1597–1610. doi:10.1056/NEJMra2311057 38692293

[3] Rajasingham R, Govender NP, Jordan A, Loyse A, Shroufi A, Denning DW, Meya DB, Chiller TM, Boulware DR. 2022. The global burden of HIV-associated cryptococcal infection in adults in 2020: a modelling analysis. Lancet Infect Dis 22:1748–1755. doi:10.1016/S1473-3099(22)00499-6 36049486 PMC9701154

[4] Chang CC, Harrison TS, Bicanic TA, Chayakulkeeree M, Sorrell TC, Warris A, Hagen F, Spec A, Oladele R, Govender NP, et al.. 2024. Global guideline for the diagnosis and management of cryptococcosis: an initiative of the ECMM and ISHAM in cooperation with the ASM. Lancet Infect Dis 24:e495–e512. doi:10.1016/S1473-3099(23)00731-4 38346436 PMC11526416

[5] Chau TT, Mai NH, Phu NH, Nghia HD, Chuong LV, Sinh DX, Duong VA, Diep PT, Campbell JI, Baker S, Hien TT, Lalloo DG, Farrar JJ, Day JN. 2010. A prospective descriptive study of cryptococcal meningitis in HIV uninfected patients in Vietnam - high prevalence of Cryptococcus neoformans var grubii in the absence of underlying disease. BMC Infect Dis 10:199. doi:10.1186/1471-2334-10-199 20618932 PMC2910700

[6] Kiertiburanakul S, Wirojtananugoon S, Pracharktam R, Sungkanuparph S. 2006. Cryptococcosis in human immunodeficiency virus-negative patients. Int J Infect Dis 10:72–78. doi:10.1016/j.ijid.2004.12.004 16288998

[7] Marr KA, Sun Y, Spec A, Lu N, Panackal A, Bennett J, Pappas P, Ostrander D, Datta K, Zhang SX, Williamson PR, Cryptococcus Infection Network Cohort Study Working Group. 2020. A multicenter, longitudinal cohort study of cryptococcosis in human immunodeficiency virus–negative people in the United States. Clin Infect Dis 70:252–261. doi:10.1093/cid/ciz193 30855688 PMC6938979

[8] Ahuja SK, Manoharan MS, Lee GC, McKinnon LR, Meunier JA, Steri M, Harper N, Fiorillo E, Smith AM, Restrepo MI, et al.. 2023. Immune resilience despite inflammatory stress promotes longevity and favorable health outcomes including resistance to infection. Nat Commun 14:3286. doi:10.1038/s41467-023-38238-6 37311745 PMC10264401

[9] Pappas PG. 2013. Cryptococcal infections in non-HIV-infected patients. Trans Am Clin Climatol Assoc 124:61–79. 23874010 PMC3715903

[10] Ueno K, Yanagihara N, Shimizu K, Miyazaki Y. 2020. Vaccines and protective immune memory against cryptococcosis. Biol Pharm Bull 43:230–239. doi:10.1248/bpb.b19-00841 32009111

[11] Neuville S, Lortholary O, Dromer F. 2000. Do kinetics of the humoral response to Cryptococcus neoformans proteins during murine cryptococcosis reflect outcome? Infect Immun 68:3724–3726. doi:10.1128/IAI.68.6.3724-3726.2000 10816535 PMC97666

[12] Dromer F, Aucouturier P, Clauvel JP, Saimot G, Yeni P. 1988. Cryptococcus neoformans antibody levels in patients with AIDS. Scand J Infect Dis 20:283–285. doi:10.3109/00365548809032452 3043650

[13] Garcia-Hermoso D, Janbon G, Dromer F. 1999. Epidemiological evidence for dormant Cryptococcus neoformans infection. J Clin Microbiol 37:3204–3209. doi:10.1128/JCM.37.10.3204-3209.1999 10488178 PMC85528

[14] Alemayehu T, Ayalew S, Buzayehu T, Daka D. 2020. Magnitude of Cryptococcosis among HIV patients in sub-Saharan Africa countries: a systematic review and meta-analysis. Afr Health Sci 20:114–121. doi:10.4314/ahs.v20i1.16 33402899 PMC7750036

[15] Lee YC, Wang JT, Sun HY, Chen YC. 2011. Comparisons of clinical features and mortality of cryptococcal meningitis between patients with and without human immunodeficiency virus infection. J Microbiol Immunol Infect 44:338–345. doi:10.1016/j.jmii.2010.08.011 21524972

[16] Gushiken AC, Saharia KK, Baddley JW. 2021. Cryptococcosis. Infect Dis Clin North Am 35:493–514. doi:10.1016/j.idc.2021.03.012 34016288

[17] Blankson JN. 2010. Control of HIV-1 replication in elite suppressors. Discov Med 9:261–266. 20350494

[18] Perfect JR, Dismukes WE, Dromer F, Goldman DL, Graybill JR, Hamill RJ, Harrison TS, Larsen RA, Lortholary O, Nguyen M-H, Pappas PG, Powderly WG, Singh N, Sobel JD, Sorrell TC. 2010. Clinical practice guidelines for the management of cryptococcal disease: 2010 update by the infectious diseases society of America. Clin Infect Dis 50:291–322. doi:10.1086/649858 20047480 PMC5826644

[19] Paccoud O, Desnos-Ollivier M, Persat F, Demar M, Boukris-Sitbon K, Bellanger A-P, Bonhomme J, Bonnal C, Botterel F, Bougnoux M-E, et al.. 2024. Features of cryptococcosis among 652 HIV-seronegative individuals in France: a cross-sectional observational study (2005-2020). Clin Microbiol Infect 30:937–944. doi:10.1016/j.cmi.2024.03.031 38556212

[20] Person AK, Crabtree-Ramirez B, Kim A, Veloso V, Maruri F, Wandeler G, Fox M, Moore R, John Gill M, Imran D, Van Nguyen K, Nalitya E, Muyindike W, Shepherd BE, McGowan CC. 2023. Cryptococcal meningitis and clinical outcomes in persons with human immunodeficiency virus: a global view. Clin Infect Dis 76:2116–2125. doi:10.1093/cid/ciad076 36821489 PMC10273391

[21] Caballero Van Dyke MC, Wormley FL Jr. 2018. A call to arms: quest for a cryptococcal vaccine. Trends Microbiol 26:436–446. doi:10.1016/j.tim.2017.10.002 29103990 PMC5910246

[22] Oliveira LVN, Wang R, Specht CA, Levitz SM. 2021. Vaccines for human fungal diseases: close but still a long way to go. NPJ Vaccines 6:33. doi:10.1038/s41541-021-00294-8 33658522 PMC7930017

[23] Poland GA, Kennedy RB. 2022. Vaccine safety in an era of novel vaccines: a proposed research agenda. Nat Rev Immunol 22:203–204. doi:10.1038/s41577-022-00695-3 35197577 PMC8864453

[24] Nayak SP, Bagchi B, Roy S. 2022. Effects of immunosuppressants on T-cell dynamics: understanding from a generic coarse-grained immune network model. J Biosci 47:70. doi:10.1007/s12038-022-00312-4 36503907 PMC9734612

[25] Alnaimat F, Sweis JJG, Jansz J, Modi Z, Prasad S, AbuHelal A, Vagts C, Hanson HA, Ascoli C, Novak RM, Papanikolaou IC, Rubinstein I, Sweiss N. 2023. Vaccination in the era of immunosuppression. Vaccines (Basel) 11:1446. doi:10.3390/vaccines11091446 37766123 PMC10537746

[26] See KC. 2022. Vaccination for the prevention of infection among immunocompromised patients: a concise review of recent systematic reviews. Vaccines (Basel) 10:800. doi:10.3390/vaccines10050800 35632555 PMC9144891

[27] Saylor K, Gillam F, Lohneis T, Zhang C. 2020. Designs of antigen structure and composition for improved protein-based vaccine efficacy. Front Immunol 11:283. doi:10.3389/fimmu.2020.00283 32153587 PMC7050619

[28] Bugya Z, Prechl J, Szénási T, Nemes É, Bácsi A, Koncz G. 2021. Multiple levels of immunological memory and their association with vaccination. Vaccines (Basel) 9:174. doi:10.3390/vaccines9020174 33669597 PMC7922266

[29] Kulshrestha A, Gupta P. 2023. Secreted aspartyl proteases family: a perspective review on the regulation of fungal pathogenesis. Future Microbiol 18:295–309. doi:10.2217/fmb-2022-0143 37097060

[30] Tarcha EJ, Basrur V, Hung CY, Gardner MJ, Cole GT. 2006. A recombinant aspartyl protease of Coccidioides posadasii induces protection against pulmonary coccidioidomycosis in mice. Infect Immun 74:516–527. doi:10.1128/IAI.74.1.516-527.2006 16369008 PMC1346669

[31] Specht CA, Lee CK, Huang H, Hester MM, Liu J, Luckie BA, Torres Santana MA, Mirza Z, Khoshkenar P, Abraham A, Shen ZT, Lodge JK, Akalin A, Homan J, Ostroff GR, Levitz SM. 2017. Vaccination with recombinant Cryptococcus proteins in glucan particles protects mice against cryptococcosis in a manner dependent upon mouse strain and cryptococcal species. mBio 8:e01872-17. doi:10.1128/mBio.01872-17 29184017 PMC5705919

[32] Specht CA, Homan EJ, Lee CK, Mou Z, Gomez CL, Hester MM, Abraham A, Rus F, Ostroff GR, Levitz SM. 2021. Protection of mice against experimental cryptococcosis by synthesized peptides delivered in glucan particles. mBio 13:e0336721. doi:10.1128/mbio.03367-21 35089095 PMC8725579

[33] Romani L, Puccetti P. 2007. Controlling pathogenic inflammation to fungi. Expert Rev Anti Infect Ther 5:1007–1017. doi:10.1586/14787210.5.6.1007 18039084

[34] Zhao T, Cai Y, Jiang Y, He X, Wei Y, Yu Y, Tian X. 2023. Vaccine adjuvants: mechanisms and platforms. Signal Transduct Target Ther 8:283. doi:10.1038/s41392-023-01557-7 37468460 PMC10356842

[35] Reyes C, Patarroyo MA. 2023. Adjuvants approved for human use: what do we know and what do we need to know for designing good adjuvants? Eur J Pharmacol 945:175632. doi:10.1016/j.ejphar.2023.175632 36863555

[36] Oleszycka E, McCluskey S, Sharp FA, Muñoz-Wolf N, Hams E, Gorman AL, Fallon PG, Lavelle EC. 2018. The vaccine adjuvant alum promotes IL-10 production that suppresses Th1 responses. Eur J Immunol 48:705–715. doi:10.1002/eji.201747150 29349774

[37] Tomljenovic L, Shaw CA. 2011. Aluminum vaccine adjuvants: are they safe? Curr Med Chem 18:2630–2637. doi:10.2174/092986711795933740 21568886

[38] Boniche C, Rossi SA, Kischkel B, Barbalho FV, Moura ÁND, Nosanchuk JD, Travassos LR, Taborda CP. 2020. Immunotherapy against systemic fungal infections based on monoclonal antibodies. J Fungi (Basel) 6:31. doi:10.3390/jof6010031 32121415 PMC7151209

[39] Spellberg B. 2011. Vaccines for invasive fungal infections. F1000 Med Rep 3:13. doi:10.3410/M3-13 21876719 PMC3155210

[40] Cassone A. 2008. Fungal vaccines: real progress from real challenges. Lancet Infect Dis 8:114–124. doi:10.1016/S1473-3099(08)70016-1 18222162

[41] Taborda CP, Nosanchuk JD. 2017. Editorial: vaccines, immunotherapy and new antifungal therapy against fungi: updates in the new frontier. Front Microbiol 8:1743. doi:10.3389/fmicb.2017.01743 28951730 PMC5599784

[42] Casadevall A, Dadachova E, Pirofski L. 2004. Passive antibody therapy for infectious diseases. Nat Rev Microbiol 2:695–703. doi:10.1038/nrmicro974 15372080

[43] Romani L. 2011. Immunity to fungal infections. Nat Rev Immunol 11:275–288. doi:10.1038/nri2939 21394104

[44] Dromer F, Charreire J, Contrepois A, Carbon C, Yeni P. 1987. Protection of mice against experimental cryptococcosis by anti-Cryptococcus neoformans monoclonal antibody. Infect Immun 55:749–752. doi:10.1128/iai.55.3.749-752.1987 3546140 PMC260405

[45] Casadevall A, Cleare W, Feldmesser M, Glatman-Freedman A, Goldman DL, Kozel TR, Lendvai N, Mukherjee J, Pirofski LA, Rivera J, Rosas AL, Scharff MD, Valadon P, Westin K, Zhong Z. 1998. Characterization of a murine monoclonal antibody to Cryptococcus neoformans polysaccharide that is a candidate for human therapeutic studies. Antimicrob Agents Chemother 42:1437–1446. doi:10.1128/AAC.42.6.1437 9624491 PMC105619

[46] Larsen RA, Pappas PG, Perfect J, Aberg JA, Casadevall A, Cloud GA, James R, Filler S, Dismukes WE. 2005. Phase I evaluation of the safety and pharmacokinetics of murine-derived anticryptococcal antibody 18B7 in subjects with treated cryptococcal meningitis. Antimicrob Agents Chemother 49:952–958. doi:10.1128/AAC.49.3.952-958.2005 15728888 PMC549259

[47] Rachini A, Pietrella D, Lupo P, Torosantucci A, Chiani P, Bromuro C, Proietti C, Bistoni F, Cassone A, Vecchiarelli A. 2007. An anti-beta-glucan monoclonal antibody inhibits growth and capsule formation of Cryptococcus neoformans in vitro and exerts therapeutic, anticryptococcal activity in vivo. Infect Immun 75:5085–5094. doi:10.1128/IAI.00278-07 17606600 PMC2168274

[48] Rosas AL, Nosanchuk JD, Casadevall A. 2001. Passive immunization with melanin-binding monoclonal antibodies prolongs survival of mice with lethal Cryptococcus neoformans infection. Infect Immun 69:3410–3412. doi:10.1128/IAI.69.5.3410-3412.2001 11292764 PMC98300

[49] Datta K, Hamad M. 2015. Immunotherapy of fungal infections. Immunol Invest 44:738–776. doi:10.3109/08820139.2015.1093913 26575463

[50] Xander P, Vigna AF, Feitosa LDS, Pugliese L, Bailão AM, Soares C de A, Mortara RA, Mariano M, Lopes JD. 2007. A surface 75-kDa protein with acid phosphatase activity recognized by monoclonal antibodies that inhibit Paracoccidioides brasiliensis growth. Microbes Infect 9:1484–1492. doi:10.1016/j.micinf.2007.08.001 17913543

[51] Matos Baltazar L, Nakayasu ES, Sobreira TJP, Choi H, Casadevall A, Nimrichter L, Nosanchuk JD. 2016. Antibody binding alters the characteristics and contents of extracellular vesicles released by Histoplasma capsulatum. mSphere 1:e00085-15. doi:10.1128/mSphere.00085-15 PMC4894687 27303729

[52] Baltazar LM, Zamith-Miranda D, Burnet MC, Choi H, Nimrichter L, Nakayasu ES, Nosanchuk JD. 2018. Concentration-dependent protein loading of extracellular vesicles released by Histoplasma capsulatum after antibody treatment and its modulatory action upon macrophages. Sci Rep 8:8065. doi:10.1038/s41598-018-25665-5 29795301 PMC5966397

[53] Almeida F, Wolf JM, Casadevall A. 2015. Virulence-associated enzymes of Cryptococcus neoformans. Euk Cell 14:1173–1185. doi:10.1128/EC.00103-15 PMC4664877 26453651

[54] Strohl WR. 2014. Antibody discovery: sourcing of monoclonal antibody variable domains. Curr Drug Discov Technol 11:3–19. doi:10.2174/1570163810666131120150043 24168292

[55] Rudkin FM, Raziunaite I, Workman H, Essono S, Belmonte R, MacCallum DM, Johnson EM, Silva LM, Palma AS, Feizi T, Jensen A, Erwig LP, Gow NAR. 2018. Single human B cell-derived monoclonal anti-Candida antibodies enhance phagocytosis and protect against disseminated candidiasis. Nat Commun 9:5288. doi:10.1038/s41467-018-07738-1 30538246 PMC6290022

[56] Nosanchuk JD, Dadachova E. 2011. Radioimmunotherapy of fungal diseases: the therapeutic potential of cytocidal radiation delivered by antibody targeting fungal cell surface antigens. Front Microbiol 2:283. doi:10.3389/fmicb.2011.00283 22275913 PMC3257868

[57] Shende R, Wong SSW, Rapole S, Beau R, Ibrahim-Granet O, Monod M, Gührs K-H, Pal JK, Latgé J-P, Madan T, Aimanianda V, Sahu A. 2018. Aspergillus fumigatus conidial metalloprotease Mep1p cleaves host complement proteins. J Biol Chem 293:15538–15555. doi:10.1074/jbc.RA117.001476 30139746 PMC6177592

